# Combining traceological analysis and ZooMS on Early Neolithic bone artefacts from the cave of Coro Trasito, NE Iberian Peninsula: Cervidae used equally to Caprinae

**DOI:** 10.1371/journal.pone.0306448

**Published:** 2024-07-10

**Authors:** Jakob Hansen, Alejandro Sierra, Sergi Mata, Ermengol Gassiot Ballbè, Javier Rey Lanaspa, Frido Welker, Maria Saña Seguí, Ignacio Clemente Conte

**Affiliations:** 1 Departament de Prehistòria, Universitat Autònoma de Barcelona, Bellaterra, Barcelona, Spain; 2 Globe Institute, University of Copenhagen, Copenhagen, Denmark; 3 Departamento de Educación, Cultura y Deporte, Diputación General de Aragón, Zaragoza, Aragón, Spain; 4 Departamento de Arqueología y Antropología, Institución Milá y Fontanals de Estudios en Humanidades (IMF), del Consejo Superior de Investigaciones Científicas (CSIC), Barcelona, Barcelona, Spain; Goethe University Frankfurt: Goethe-Universitat Frankfurt am Main, GERMANY

## Abstract

Few studies have combined the analysis of use-wear traces, traceology, and the proteomic taxonomic identification method Zooarchaeology by Mass Spectrometry (ZooMS). Traceology provides information on the usage, in this case, of bone artefacts, while ZooMS allows for taxonomic identifications where diagnostic features are otherwise gone. The approaches therefore offer complementary information on bone artefacts, allowing for insights into species selection strategies in bone tool manufacture and their subsequent use. Here we present a case study of 20 bone artefacts, mainly bone points, from the Early Neolithic cave site of Coro Trasito located on the southern slope of the Central Pyrenees. Hitherto, studies on Early Neolithic bone artefacts from the Iberian Peninsula have suggested based on morphological assessments that *Ovis aries*/*Capra hircus* constituted the majority of the bone material selected for bone tool production. However, the taxonomic identification in this study suggests that, at this site, Cervidae was selected equally to that of *O*. *aries*/*C*. *hircus*. Furthermore, bone artefacts made from Cervidae specimens seem to be utilised in a wider range of artefact types compared to *O*. *aries*/*C*. *hircus*. Coro Trasito’s bone artefact species composition is probably site-specific to some degree, however, morphological assessments of bone artefacts might not be representative and could be biased towards certain species. Therefore, research on bone artefacts’ usage could possibly gain new insights by implementing ZooMS in combination with traceology.

## Introduction

Numerous disciplines in archaeological research have been emerging, growing, and evolving in at least the past 250 years [1]. These applications on both ancient organic and inorganic materials have been manifested through, among others, the earliest chemical analysis of archaeological bronze artefacts in 1777 [2], the incredibly impactful development of radiocarbon dating in the late 1940s [3, 4], the study of animal remains termed zooarchaeology around 1970, though having its roots in the nineteenth century [5], and later stable isotope and aDNA analyses in the late twentieth century [6, 7]. Archaeological research is thus a multitude of specialised disciplines. However, cross-disciplinary thinking, reaching beyond combining the term archaeology with one specialised method, has the potential to extract additional information from the archaeological record.

In this study, we explore the interface between the two disciplines of traceology and palaeoproteomics on bone artefacts uncovered from the Early Neolithic site of Coro Trasito, located in the Central Pyrenees. Traceology, or use-wear analysis, can be traced back to around 1900, though being more widely employed after the 1950s [8, 9]. Such analyses interpret the functionality of archaeological objects [9–11] and, in extension, technology and resource exploitation as well as transport and taphonomic alterations [12, 13]. Traceological analyses are mostly based on experimental and ethnographic data of micro and macro use-wear traces and residue remains of various materials, e.g. ceramics, bones, and lithics [9]. The study of ancient proteins, or palaeoproteomics, has its origins in the 1950s with the detection of amino acids in fossils [14]. The approach experienced a major leap in the early 2000s with the implementation of soft-ionization methods in protein mass spectrometry, allowing for the retrieval of partial, and sometimes indirect, protein sequence data [15, 16]. Palaeoproteomics thus has the potential to, e.g., study dietary practices [17], evolutionary and phylogenetic relations exceeding deep time barriers not possible with aDNA [18, 19], and retrieve taxonomic identifications of various organic materials where morphological diagnostic features are not available [20–23].

Both traceological and palaeoproteomic approaches have been applied to bone artefacts from a range of time periods. Since they provide complementary information on the use of artefacts, the existing, and limited, amount of studies conducted on single sets of bone artefacts [24–27] is in need of being expanded. The convergence between these two areas of application has already shown great scientific promise. Therefore, the 20 Early Neolithic bone artefacts from the Cave of Coro Trasito were taxonomically identified through Zooarchaeology by Mass Spectrometry (ZooMS) [28] which is a peptide mass fingerprinting (PMF) method based on the presence of type I collagen (COL1). Furthermore, the traceological analyses conducted specified the functions of artefacts and which materials the artefacts were utilised in relation to. It was therefore possible to assess the species chosen for the production of certain types of bone tools at the site of Coro Trasito on a more explicit level.

Here, we found that based on the artefacts successfully taxonomically identified, Caprinae and Cervidae were evenly identified among those. Regarding selection strategies involved in the bone tool manufacture, Caprinae was selected for the production of artefacts related to the processing of vegetation. Hereof *Capra* sp. bone points tended towards being used in relation to bark, while Caprinae (not *Capra* sp.), most like *Ovis aries*, was possibly used in weaving plant fibres. Cervidae was however used in a comparably broader range of artefact categories entailing projectile points, points used in relation to plant fibres, and one indeterminate usage.

## Material and methods

### The site of Coro Trasito

**Fig 1:**
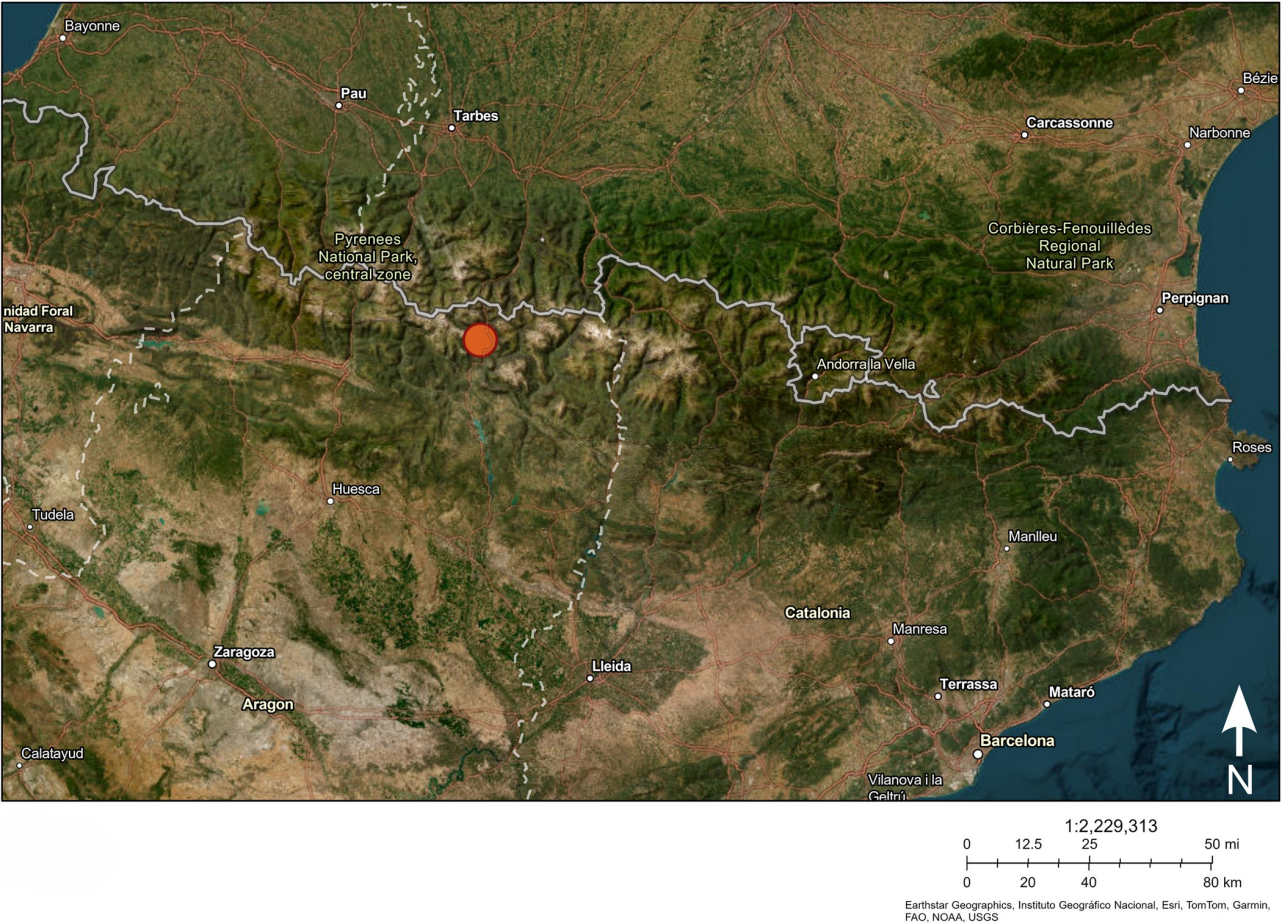
The location of Coro Trasito is indicated by the orange dot. Map created using USGS ArcGIS Online Map Viewer.

The cave of Coro Trasito is located on the southern slope of the Pyrenees in the municipality of Tella-Sin (Huesca, Spain) at an altitude of 1,548 m a.s.l. [29] (lat.: 42.595587, long.: 0.177980) (Fig 1). The site was discovered in modern times and surveyed in the 1970s, with findings of ceramics and fragments from grinding stones, among others [29, 30]. Excavations were resumed in 2011, 2013, and almost continuously between 2014 and 2021 [31].

During the past ten years ceramics, lithics, bone artefacts, flora and fauna, and structures of various functions have been unearthed. Typologically, the cultural layers could be dated to the Early Neolithic and Bronze Age, as confirmed by radiocarbon dating [29, 30, 32]. Concerning the Neolithic strata, various phases or occupation patterns have been suggested [32–34], nevertheless, anthropic activities have been dated to approximately 5,300 to 4,400/4,360 cal BCE.

**Fig 2:**
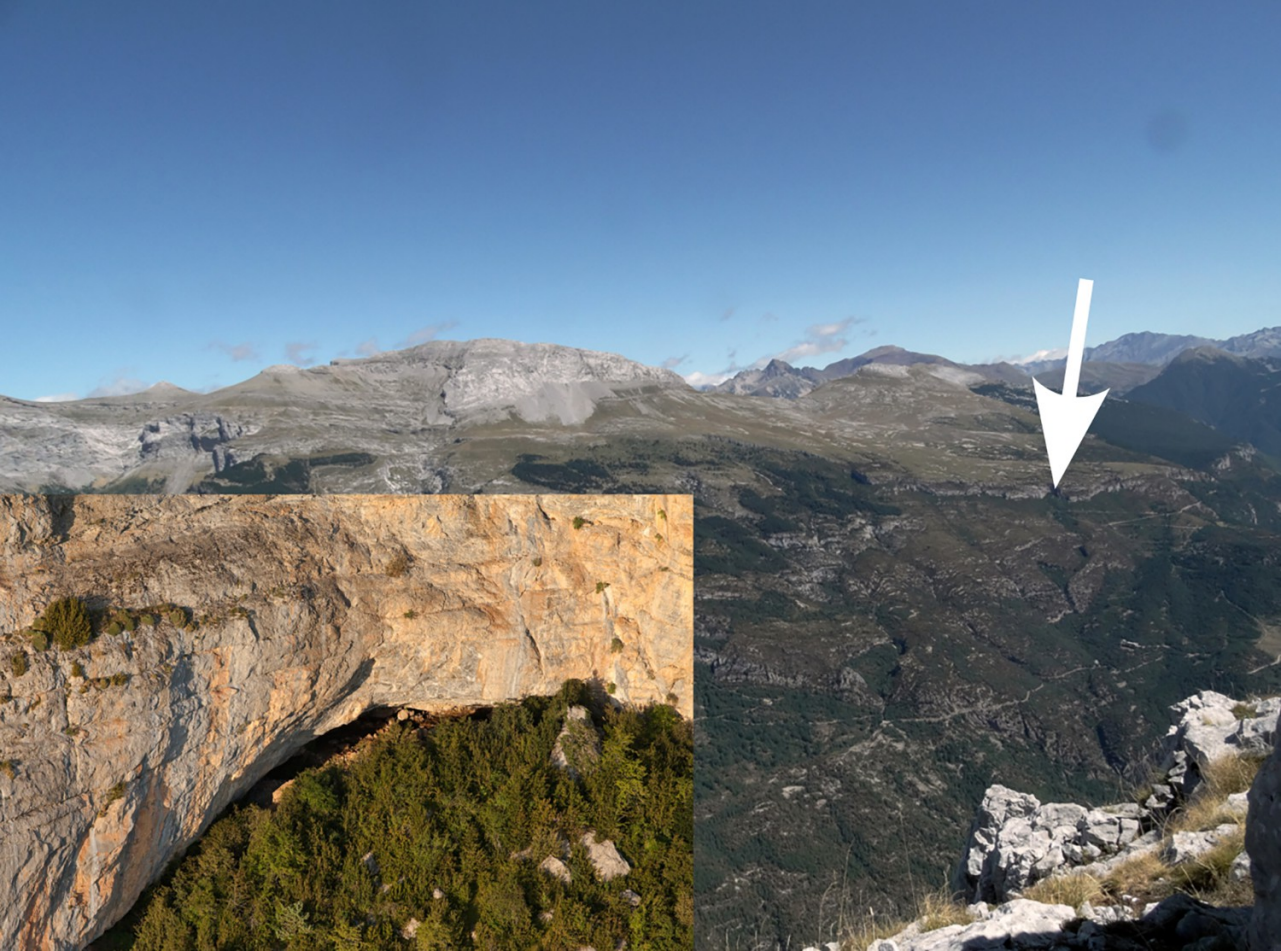
The cave of Coro Trasito. The arrow points to the location of the close up of Coro Trasito seen in the lower left corner.

Though the site is located on the upper fringe of the montane stage (Fig 2), Coro Trasito shows evidence of broad agricultural economic practices and continuous occupation during the Early Neolithic [35, 36]. This challenges the thought of lowlands having a broad spectrum of economic strategies and mountainous areas being strictly used for specialised herding practices [36].

**Fig 3:**
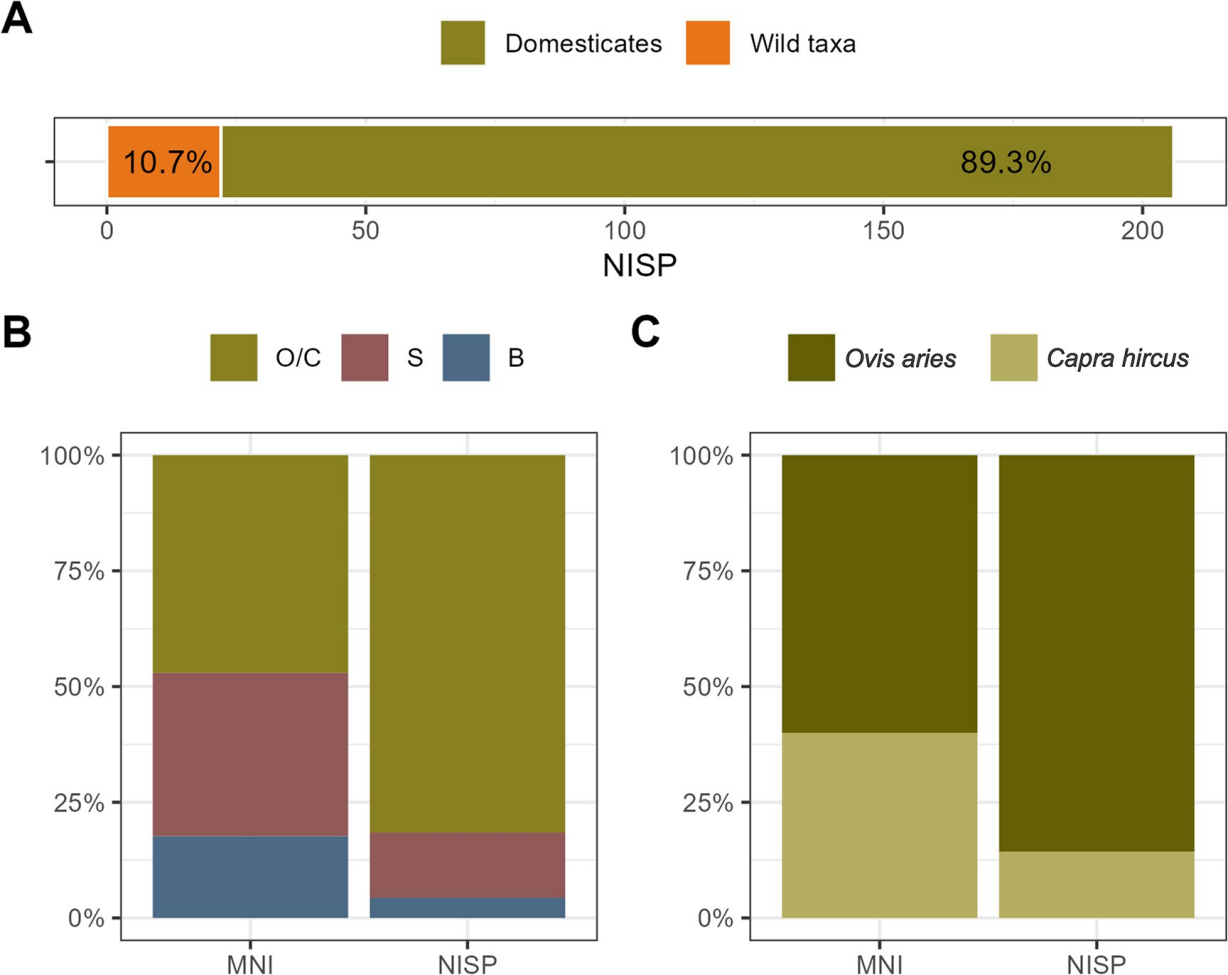
Faunal remains from Coro Trasito from layers referred to as NEO II, dating to approximately 4,900–4,700 cal BCE [34]. (A) Domesticated (n = 184) and wild taxa (n = 22). (B) *O*. *aries*/*C*. *hircus* (O/C); MNI = 8 | NISP = 150, *S*. *domesticus* (S); MNI = 6 | NISP = 26, *B*. *taurus*. (C); MNI = 3 | NISP = 8. C) *O*. *aries*; MNI = 3 | NISP = 12, *C*. *hircus*; MNI = 2 | NISP = 2. The MNI and NISP counts stem from the *O*. *aries*/*C*. *hircus* portion shown in panel B.

The faunal remains corresponding to the chronology focused on in this study, approximately 4,900–4,700 cal BCE, [34, 37, 38] reveal a significant dominance of domestic taxa including *Ovis aries*/*Capra hircus*, *Sus domesticus*, and *Bos taurus* (Fig 3A). Among these, the *O*. *aries*/*C*. *hircus* stands out as the most dominant domesticate (Fig 3B). Based on the NISP, 9.33% could be determined as either *C*. *hircus* or *O*. *aries*, the latter constituting the largest portion (Fig 3C) (S1 Table).

Slaughter patterns for the domesticates reveal that *O*. *aries*/*C*. *hircus* were mainly slaughtered between 12 and 24 months old, *S*. *domesticus* between 18 and 21 months old and *B*. *taurus* over 24 months old, all suggesting a source of meat, fat, and/or marrow. However, the presence of neonate and infantile *O*. *aries*/*C*. *hircus* individuals (MNI = 3 out of 8) may suggest the cave was utilised as a stable and/or breeding area [39]. The exploitation of dairy was further confirmed by milk residues documented in ceramic pots [40]. Taphonomic analyses also reveal a significant number of remains with alterations due to their deposition in manure, which may be related to the cohabitation of animals and humans in the same space [39]. Among the wild taxa (Fig 3A), the presence of *Cervus elaphus* (NISP = 10; MNI = 1), *Capreolus capreolus* (NISP = 2; MNI = 2), *Capra pyrenaica* (NISP = 2; MNI = 1), *Sus scrofa* (NISP = 1; MNI = 1), *Oryctolagus cuniculus* (NISP = 5; MNI = 1), *Vulpes vulpes* (NISP = 1; MNI = 1) and *Ursus arctos* (NISP = 1; MNI = 1) is documented, all wild animals being over two years old.

At Coro Trasito, various livestock management practices are moreover documented through stable isotope analyses, suggesting different grazing strategies. Wild herbivores have seemingly low and homogeneous nitrogen (δ^15^N) values compared to *O*. *aries*, *S*. *domesticus*, and *B*. *taurus*. However, *C*. *hircus* has similar low δ^15^N levels to wild herbivores, which could indicate them grazing in environments less affected by humans, e.g., areas not employed for agricultural practices. This could in turn suggest that *O*. *aries* and *B*. *taurus* had access to cultivated fields or the supply of crop surpluses based on the enriched δ^15^N values [34].

Carpologically, agricultural practices have also been documented via the cultivated plant remains recovered. This includes the chaff and grains of naked barley (*Hordeum vulgare var*. *nudum*) and naked wheat (*Triticum aestivum s*.*l*./*durum Desf*./*turgidum* L.), alongside potential field weeds like *Bromus* sp., *Polygonum convolvulus*, *Galium aparine*, and *Chenopodium* sp. [33]. Pollen analysis also indicates the presence of cultivated fields in the vicinity of the cave [36, 41].

The complex strategies involved in husbandry, both for animals and plants, gathering of autumn wild plants [33], and the evidence of storage pits [30] suggest that the Cave of Coro Trasito was a somewhat permanent occupation during the Neolithic, or at least played a more complex role than that of a seasonal sheepfold for livestock.

### Sample selection

A total of 26 bone objects from the Pyrenean cave site of Coro Trasito were selected for traceological and zooarchaeological assessment, as well as taxonomic identification analysis through ZooMS [28]. No permits were required for the described study, which complied with all relevant regulations. However, six of the 26 initial specimens were excluded as three were later established as bone fragments, and the remaining three specimens are dated to the Bronze Age and were therefore deemed as being out of our research scope, which focuses on bone artefacts from the Early Neolithic. Additional information on the six excluded specimens can be found in S2 Table, but have otherwise been kept out from further analysis in the following chapters. The majority of the remaining bone artefacts assessed originate from approximately 5,000 to 4,585 cal BCE. Only one complete bone point used to weave plant fibres (ID: 11.23.S1.1009.-213 [29]) is missing from the study.

Furthermore, in addition to the selected bone artefacts subjected to traceological analysis and ZooMS in this study, other raw materials of animal origin have been characterised as bone tools at Coro Trasito, but were possible to identify morphologically to genus or species level. Four *C*. *elaphus* antlers have been documented as three independent tools and the last one functioning as a component of another antler tool as a handle. The latter would have consisted of an intermediate piece accommodating a point, possibly used to press flint cores and extract blades as a tool holder. The other three antler tools consist of points made from the tines of an antler, one used as a retoucher in lithic knapping, another as a ’punch’ for the indirect knapping of flint blades, and the last, configured with a 45-degree angle, used as a gouge or chisel for woodworking. Furthermore, a *S*. *scrofa* tusk and mollusc shells of marine origin were present at the site. The shells were mainly used as tools for the production of ceramics [42], while the *S*. *scrofa* tusk assisted in the processing of plant fibres [43].

The bone artefacts chosen for taxonomic identification via ZooMS consist mostly of pointed and/or needle-shaped elements. Typologically, one awl, seven bone points (or awls), two needles, two spatulas, three pointed tools, two projectile points, one pendant, and two undetermined artefacts are represented.

### Experimental design

The traceological analysis was performed on the 26 specimens prior to sampling for ZooMS. In general, nitrile gloves were used and instruments and surfaces were cleaned with 1.19 M CH_3_CH_2_OH (ethanol) for the handling and sampling of the specimens. For the post sampling ZooMS procedure, instruments and surfaces were cleaned in between each sampling using 0.07 M NaOCl (sodium hypochlorite, or bleach) and ethanol, consecutively. Zooarchaeological assessments were performed sporadically throughout the study.

### Traceological analysis

Macro- and microscopic analyses were performed using equipment available at the Institución Milá y Fontanals, El Consejo Superior de Investigaciones Científicas, (IMF-CSIC). This entailed an Olympus SZ binocular magnifying glass with up to 80x magnification, and a Leica DM2500 metallographic microscope with objectives and a duplicator allowing for magnifications between 50x and 400x. The Coro Trasito specimens included in this study were compared with the reference material from the IMF-CSIC trace library consisting of both experimental and archaeological bone instruments [44–46].

### ZooMS

Each specimen was sampled with the minimally invasive approach using micro-grit polishing film sticks (PFP Polishing film, 2” Round Aluminium Oxide, PSA, Precision Fiber Products, Inc.) [47, 48]. Both sides of the sticks contained polishing film (30 μm grit size) which in 20 circular movements were applied to a fixed area of the specimen surface. It is difficult to measure the exact amount of bone powder sampled, as the polishing film is included in the tube for further processing, and is not pre-weighed to avoid cross-contamination. Nevertheless, we estimate that less than 0.5 mg of bone powder was obtained per specimen using polishing film. We chose a grit size of 30 μm as it is likely to yield more diagnostic markers compared with smaller grit sizes [49]. The sticks were then separately collected in 1.5 mL microtubes (Protein LoBind, Eppendorf).

100 μL 0.05 M NH_4_HCO_3_ (ammonium bicarbonate, hereafter AmBic) was added to the microtubes for subsequent incubation at 65°C for 1 hour using a heating block (Thermal Shake *lite*, VWR*)* [50]. After gelatinisation, 50 μL of the supernatant was transferred to a new microtube with 1 μL of 0.4 μg/μL trypsin (Promega, #V115A) and digested for 18 hours at 37°C. Digestion was stopped by adding 1 μL of 0.13 M CF_3_CO_2_H (trifluoroacetic acid). Samples were purified and desalted using a C18 Hypersep™ plate (Thermo Fisher) and spotted on an MTP 384 target MALDI plate ground steel BC (Bruker) in triplicates. Spots comprised of 1 μL eluted peptides and 1 μL of α-cyano-4-hydroxycinnamic acid (CHCA) matrix solution [18].

Mass spectra were acquired using two mass spectrometers. First, all samples were run through a Bruker UltrafleXtreme MALDI-ToF MS in reflector mode, positive polarity, set to a laser intensity of 50–70%, and a mass range of 799–4,000 m/z. Each sample was externally calibrated against adjacent spots containing a mixture of six peptides (des-Arg1 Bradykin m/z = 904.681, Angiotensin I m/z = 1295.685, Glu1-Fibrino- peptide B m/z = 1750.677, ACTH (1–17 clip) m/z = 2093.086, ACTH (18–39 clip) m/z = 2465.198 and ACTH (7–38 clip) m/z = 3657.929). Hereafter, 14 samples of poorer quality were selected for reruns on a Bruker timsTOF fleX MALDI-tims-Q-ToF in reflector mode, positive polarity, set to a laser intensity of 40%, and a mass range of 799–4,000 m/z. Calibration was obtained for a total of 24 samples, which were calibrated against 3 spots containing the mixture of six peptides listed above.

Triplicate spectra were merged [51] and processed using *MALDIquant v*.*1*.*22*.*2* [52] and *MALDIquantForeign v*.*0*.*14*.*1* [53] through the software R [54].

### Zooarchaeological analysis

Anatomical and taxonomic identification was carried out using the reference collection of the Laboratori d’Archaeozoologia de la Universitat Autonoma de Barcelona. Specimens that could not be taxonomically identified, were classified by size group accounting for data from previous zooarchaeological studies [34]. Large mammals were considered to correspond to cattle or similar-sized species, large-medium mammals to *C*. *elaphus* or *Sus* sp., and medium mammals to *O*. *aries*, *C*. *hircus*, *Sus* sp., *Rupicapra rupicapra*, or *C*. *capreolus*. In addition, the assemblage was taphonomically analysed, with special attention to the burnt bones. For this purpose, the colour criteria of Stiner et al. [55] were followed, establishing different groups: 0) not burned, 1) slightly burned, 2) lightly burned, 3) fully carbonised, 4) less than half calcined, 5) more than half calcined, 6) fully calcined.

### Statistical analysis and data visualisation

Visualisations were mainly conducted through R software [54] using the packages *tidyverse v*.*2*.*0*.*0* [56], *dplyr v*.*1*.*1*.*4* [57], *webr v*.*0*.*1*.*5* [58], *ggpubr v*.*0*.*6*.*0* [59], *cowplot v*.*1*.*1*.*3* [60], and *scales v*.*1*.*3*.*0* [61]. An alluvial plot was created using a website intended for the purpose [62].

One significance test was performed using Excel for Microsoft 365’s T.TEST function which is based on the Student’s paired t-test [63].

## Results

### Traceological analysis

At a technological level, the analysed artefacts exhibit different degrees of surface modifications. Thus, in terms of traces from manufacturing techniques related to the production of the bone artefacts and the subsequent wear and tear of the finished products, we have been able to distinguish between the various procedures involved in producing the tools, which materials the finished tools were used against, and their possible functions.

One manufacturing technique used to reduce and smooth surfaces is visible through the traces caused by scraping with a lithic tool This is e.g. documented on fragments whose specific usage could not be determined (Fig 4.4, 4.9 and 4.4). Furthermore, abrasion on minerals, likely sandstone, primarily to shape the pointed apex, leaves deep longitudinal grooves and gloss on elevated areas, as is visible on the microtopography (Figs 4.15, 4.10, 5.2 and 5.3). In this instance, the artefacts fractured during the manufacturing, as there are no traces of use. In other cases, use-wear traces due to contact with plant fibres have altered the previous manufacturing marks, but they are still recognisable to some degree (Fig 5.5). This method of sharpening the distal end is common for most artefacts and has been identified on several distal fragments, as depicted in Fig 4.16 and 4.17, which may have fractured during use. However, once the artefacts have been used extensively, the surfaces become much more uniform. Fig 6B exhibits this through the extensive polishing, which nearly completely masks previous manufacturing traces.

**Fig 4:**
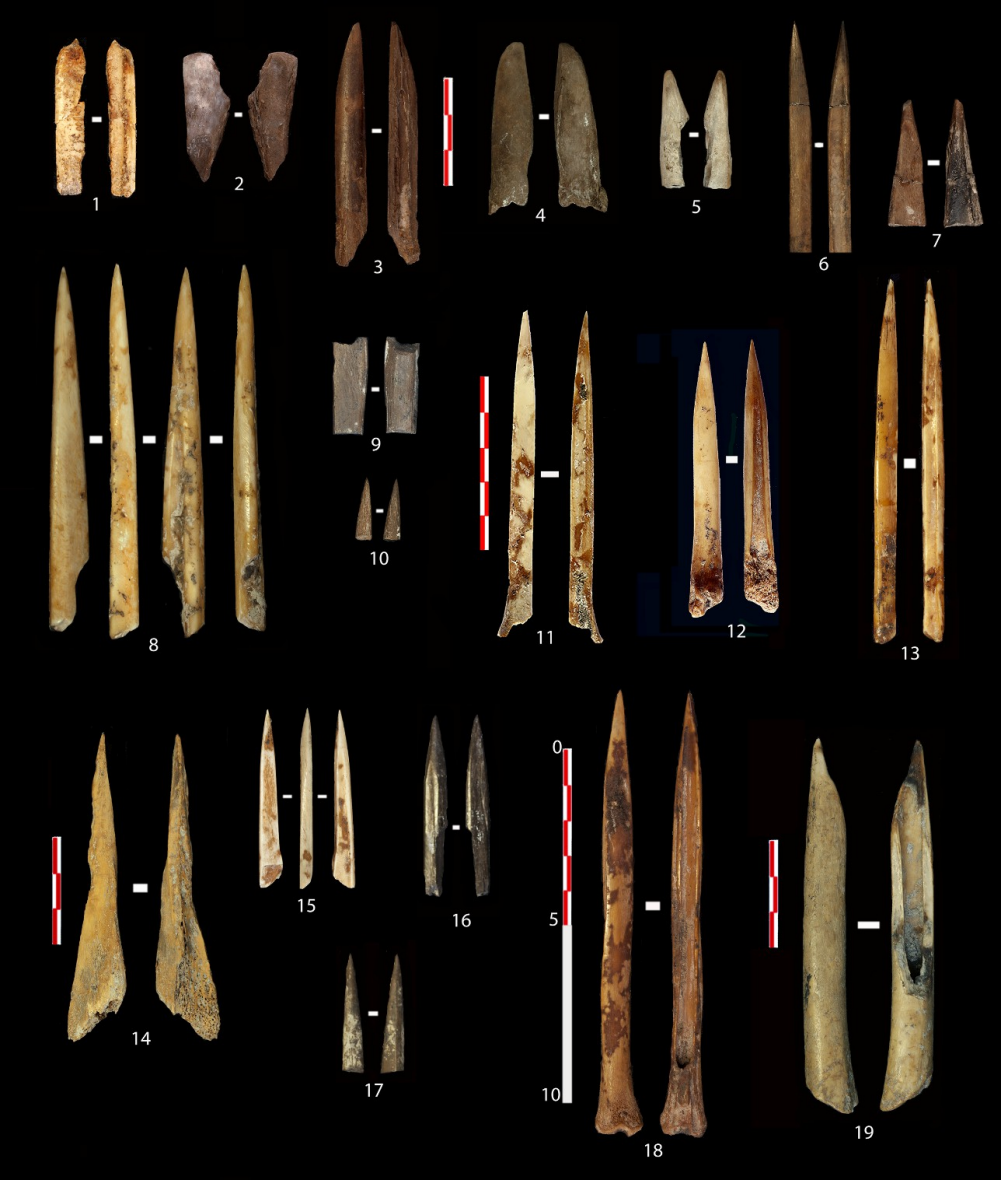
Fragments and complete bone objects from the Early Neolithic site of Coro Trasito. The ZooMS ID numbers are indicated for each specimen are as follows: (1) CT_0013. (2) CT_0021. (3) CT_0007. (4) CT_0020. (5) CT_0012. (6) CT_0006. (7) CT_0017. (8) CT_0001. (9) CT_0019. (10) CT_0024. (11) CT_0003. (12) CT_0004. (13) CT_0005. (14) CT_0002. (15) CT_0010. (16) CT_0015. (17) CT_0016. (18) CT_0011. (19) CT_0009. The pendant with ZooMS ID CT_0014 is not included in the figure. Further information is available in S2 Table.

**Fig 5:**
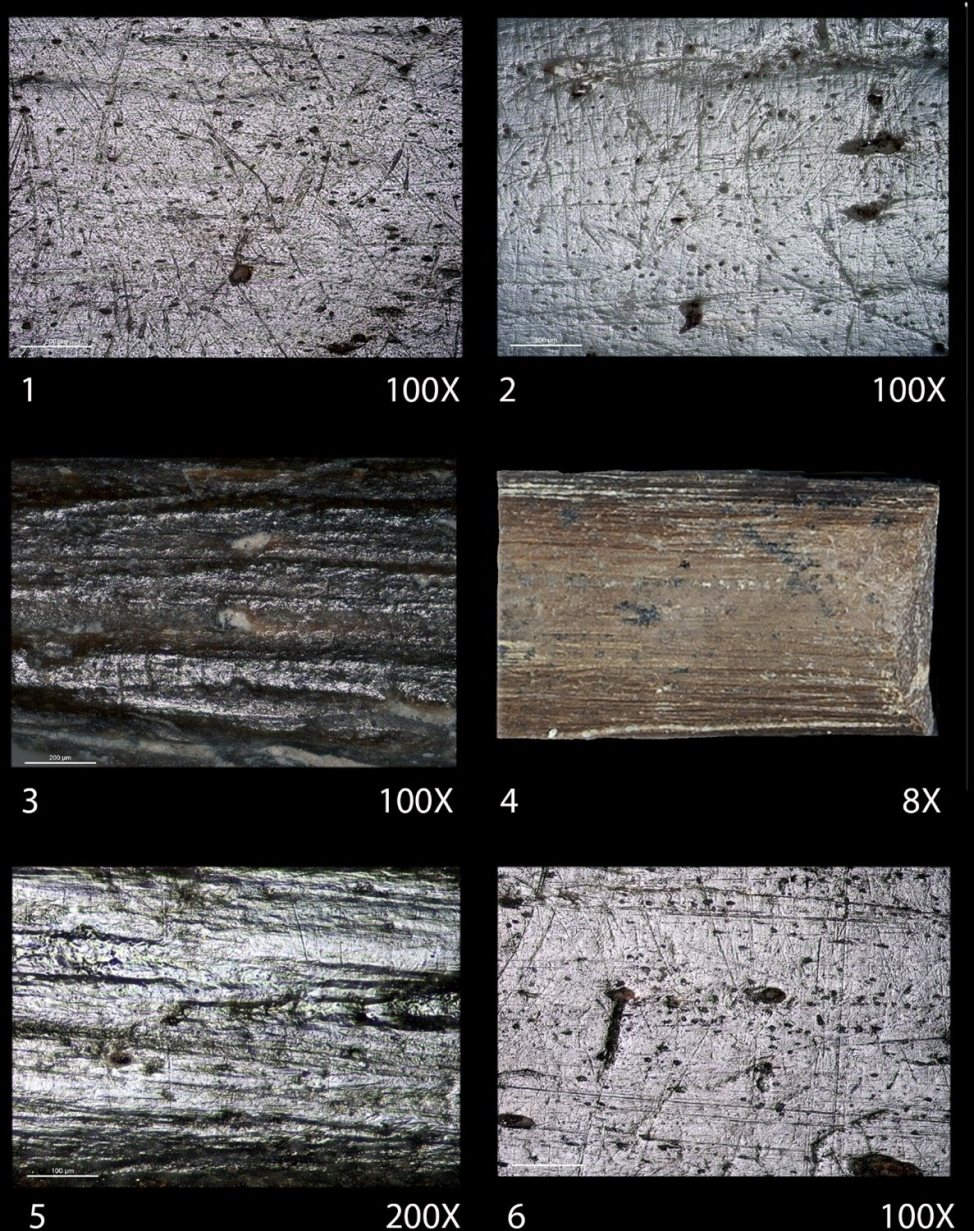
Different high-resolution images of bone object surfaces. (1) (CT_0001) and (2) (CT_0010) show a specific polish on the entire surface as a result of the technique used in their manufacture. (3) (CT_0024) Deep striations and shiny elevated areas due to abrasion with a mineral matrix to obtain a pointed morphology. (4) (CT_0019) Striations produced by scraping with a lithic instrument. (5) (CT_0005) Traces of use due to contact with plant fibres at the distal extremity. (6) (CT_0005) Reference material showing traces attributed to hand grasping in the medial/proximal area.

**Fig 6:**
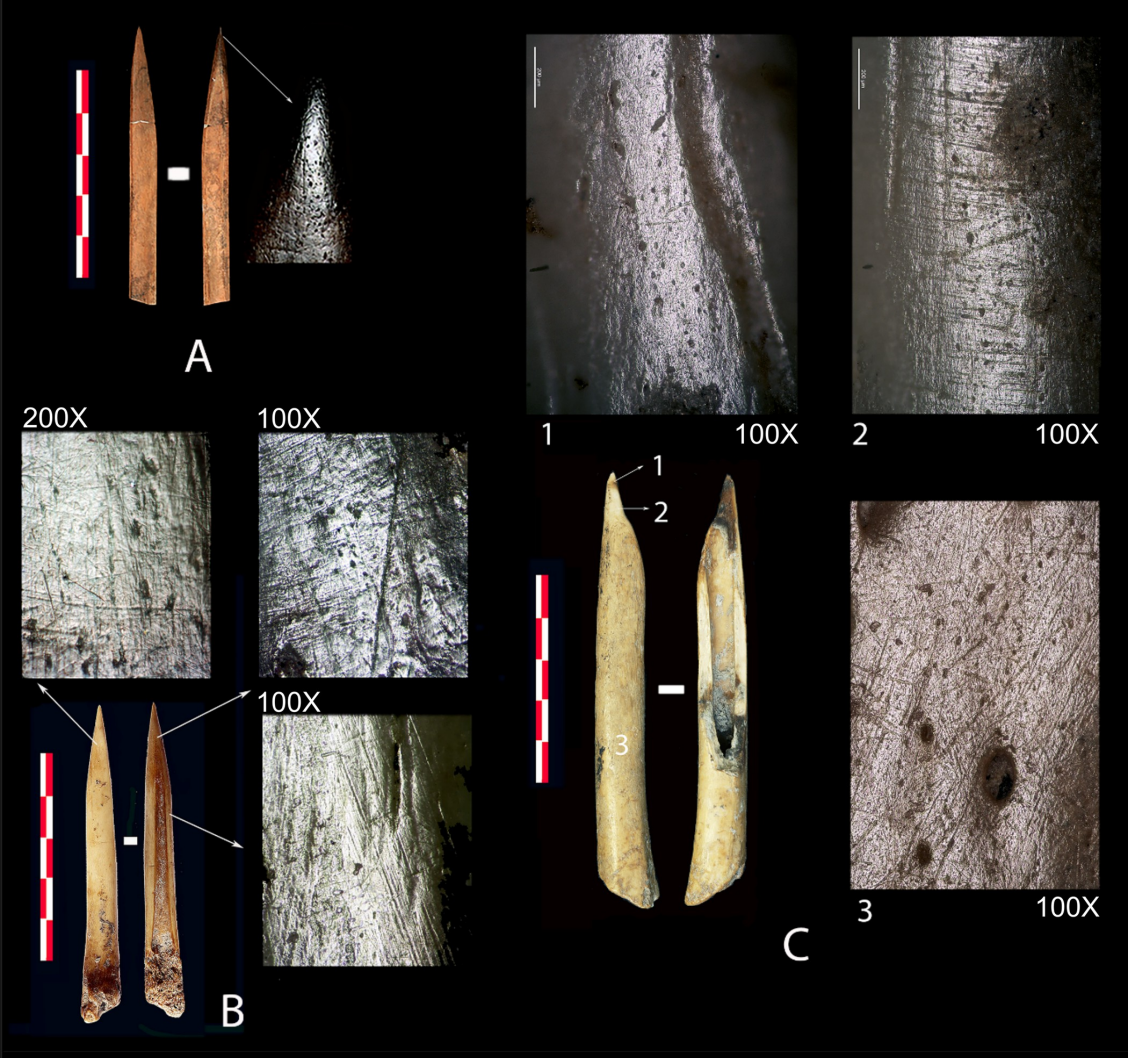
Three artefacts with multiple high-resolution images, displaying various surface modifications. (A) Needle used to pierce skin or leather (CT_0006). (B) Bone point with traces related to the work of plant fibres (weaving, basket weaving, or cordage) (CT_0004). (C) Artefact with 1; traces related to penetration, located at the apex of the tip, 2; an indication of twisting, possibly caused by friction between tool and vegetation, likely bark, and 3; traces related to gripping during utilisation of the tool (CT_0009).

Another type of surface treatment is found on three fragments, two of them pointed, indicating possible impact fractures (Fig 4.8 and 4.15) as the objects on which they are present typologically could be projectile points. The third (Fig 4.1) is completely altered due to taphonomic modifications such as carnivore biting and fractures that prevent determining its shape and function. All three have a type of polish on their entire surface displaying multiple small circular depressions accompanied by multidirectional striations of different sizes (Fig 5.1 and 5.2). This polish resembles grip traces, as seen in Fig 5.6 [29, 45, 64]. It is worth considering whether the phenomenon could be due to extensive handling by gripping or by polishing as the result of contact with leather or skin. It would also be possible that the objects were hafted and transported in a leather quiver, as they likely are arrowheads, thus acquiring the multidirectional striations as a polish. However, this would need to be confirmed via experimental studies.

In addition, specific types of traces and fractures, which are not intentional, and have influenced the external appearance and morphology of the artefacts have been recorded as well. Besides the grip traces mentioned above, there are also certain artefacts documented at Coro Trasito whose surfaces have not been prepared prior to usage. This include spatulas created from flat bone fragments that have been shaped and formed by their own use upon contact with an abrasive material such as ceramic paste [42]. The spatulas in this study are however made from long bones (Fig 4.2 and 4.4). Friction with a specific working angle on this material causes a blade to be shaped with certain morphological characteristics that give the spatula its appearance. Moreover, certain bone fragments, that due to the type of fracture inflicted have acquired an affordance or shape suitable for their direct use as an instrument, such as the tools seen in Fig 4.3 and 4.7, among others.

Altogether, the artefacts can be categorised according to typology and function based on shape and use-wear analysis as follows (see S2 Table for cross-referencing): 1) Two needles (Fig 4.6 and 4.7) and one awl (Fig 4.3) used for penetrating skin or leather. 2) Two spatulas (Fig 4.2 and 4.4) used in working with ceramic surfaces. 3) Five artefacts exhibiting specific polishing, two of which could be projectile points (Fig 4.8 and 4.15), one with no apparent use (Fig 4.1), and two classified as having an undetermined use (Fig 4.5 and 4.9). 4) A total of eight artefacts were used for transforming plant resources into consumer goods (Fig 4.11-4.14 and 4.16-4.19), whereof the two widest artefact’s tips came into contact with the material being worked (Fig 4.14 and 4.19). 5) The remaining five instruments consist of two distal fragments (Fig 4.16 and 4.17) and three complete ones (Fig 4.11-4.12 and 4.18), which were used in weaving plant fibres. The two fragments show discolouration related to thermal alteration, and it is not possible to confirm when the fracture occurred, whether during use or due to taphonomic reasons. In both cases, due to the poor development of use traces, it seems plausible that they were not utilised extensively as the manufacturing traces are still clearly recognisable. In contrast, this is not the case for the intact artefacts, which show well-developed use-wear traces at the distal tips, although two specimens (Fig 4.11 and 4.18) have dark spots on the remaining body, most likely due to sedimentary conditions corroding and altering them.

### ZooMS

**Fig 7:**
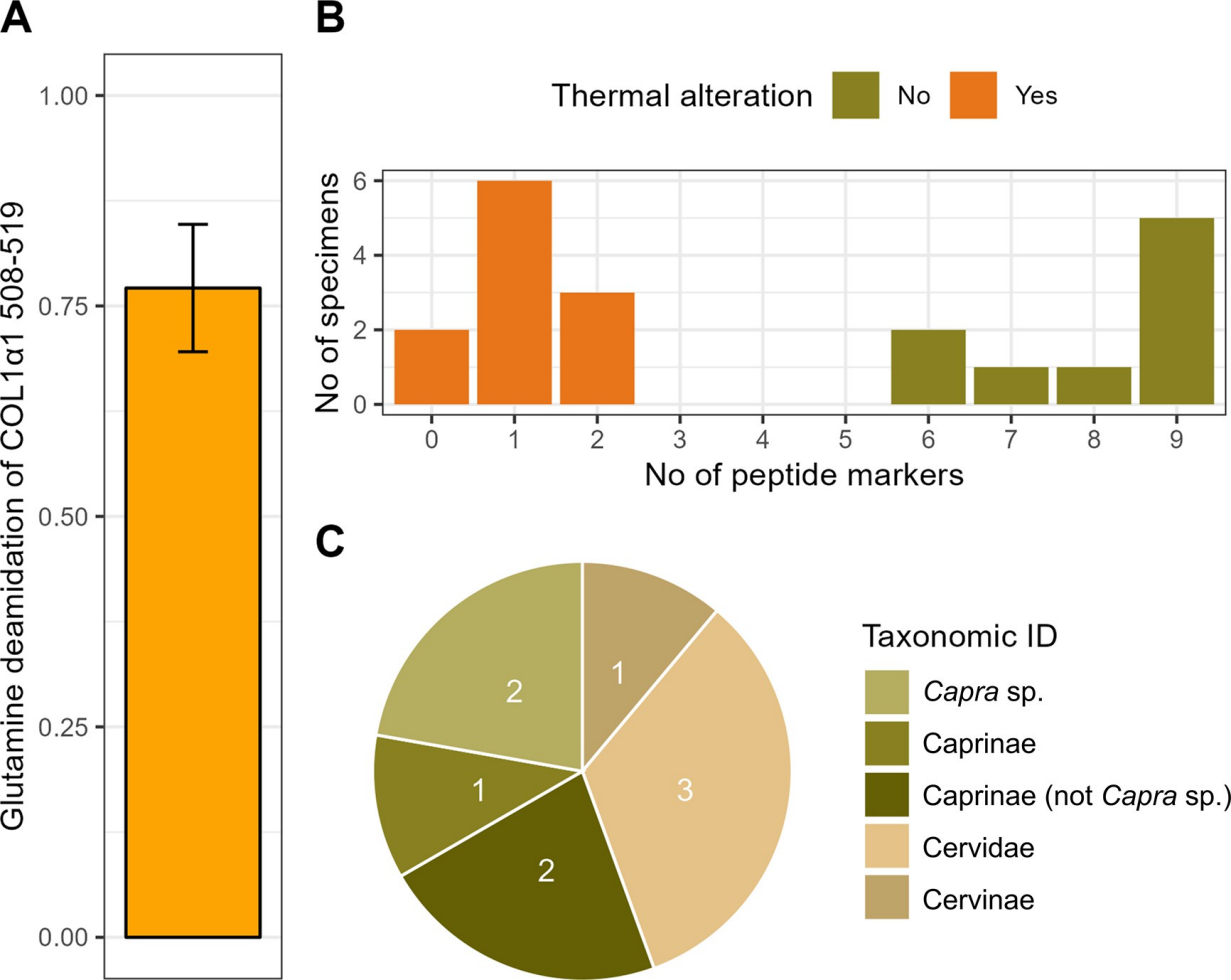
ZooMS results. (A) Glutamine deamidation of COL1α1 508–519, full deamidation indicated by 0 and no deamidation indicated by 1. (B) Count of peptide markers observed from each bone artefact (n = 20) and an indication of thermal alteration being present or absent. Peptide marker counts can range from 0 to 9 in the followed taxonomic approach. (C) Lowest taxonomic identifications possible through ZooMS (excl. indeterminate (n = 11)).

From the 20 bone artefacts sampled it was possible to taxonomically identify nine to family or genus level (S2 Table). Five samples could be identified as Caprinae, Caprinae (not *Capra* sp.), or *Capra* sp., while the remaining four could be identified as Cervidae or Cervinae (Fig 7C). In the context of the Early Holocene Pyrenees, Caprinae (not *Capra* sp.) could be either *O*. *aries* or *R*. *rupicapra*, and *Capra* sp. could be identified as *C*. *pyrenaica* or *C*. *hircus*. Cervidae refers to *C*. *elaphus* or *C*. *Capreolus* whereas Cervinae can be narrowed down to *C*. *elaphus*, since no other members of the Cervinae subfamily occur in the region at the time. Though sample sizes are low, it is worth noting the discrepancy between the low amount of wild taxa represented in the non-artefact portion (Fig 3A) and the close to fivefold increase of wild taxa in the bone artefact portion (Fig 7C), assuming Caprinae, Caprinae (not *Capra* sp.), and *Capra* sp. are domesticates.

Unlike the samples where taxonomic identifications were possible, the unidentified portion all have some degree of thermal alteration, being either slightly burned (n = 7), carbonised (n = 2), or fully calcined (n = 1) [55], and lastly, one where it was unclear if heat treatment had been implemented, though only two out of nine peptide markers could be recognised. The

Glutamine deamidation values of COL1α1 508–519 could be retrieved from eight of the nine taxonomically identified samples showing a relatively consistent deamidation level of 0.77 ± 0.08 SD (Fig 7A). Full deamidation is indicated by 0, while no deamidation is indicated by 1 [65]. Deamidation is a post-translational modification (PTM) of the amino acids glutamine to glutamic acid and asparagine to aspartic acid. Due to the slower deamidation rate of glutamine [66], glutamine deamidation is generally favoured over asparagine deamidation in archaeological studies [65]. In terms of deamidation values calculated based on ZooMS data, specifically the peptide COL1α1 508–519, which has a comparably slow deamidation rate compared with other COL1 peptides, samples reaching beyond at least 1700 years BP are likely to have deamidation levels below 0.9 [67]. MALDI-ToF MS deamidation values of the same peptide and deriving from modern and recent bone proteome extracts generally have values close to or identical to 1 [68], while in contrast Late Pleistocene bone extracts provide deamidation values for this peptides covering a wide range of values, from 0 to close to 0.9 [69–74]. This large variability is likely explained by differences in local sedimentary conditions, including the absence or presence of moving water, temperature, and mineralogical composition, among other factors [75]. In this context, the deamidation level calculated for the Coro Trasito samples suggests that the collagen peptides extracted are endogenous to the bone artefacts.

Finally, 13 out of the 20 Neolithic artefact samples were selected for reruns through a Bruker timsTOF fleX MALDI-tims-Q-ToF as it has a mass resolution of 60,000 compared to the ultrafleXtreme MALDI-ToF’s mass accuracy of 40,000 [76] in hopes of gaining additional peptide marker information. 11 samples were selected as no taxonomic identifications were established and few peptide markers were observed (1.09 ± 1.72 SD out of 9), and the remaining two samples (7 ± 1.41 SD out of 9) were chosen as the peptide marker COL1α2 757–789 was not detected which would provide a more specific taxonomic identification. Most of these samples displayed signs of thermal alteration, which is related to poor preservation of collagen, and has evidently affected the number of peptide markers observed in this study (Fig 7B). Significantly fewer peptide markers could be observed through the MALDI-tims-Q-ToF spectra (1.08 ± 2.22 SD out of 9) (Student’s paired t-test: p = 0.001), compared with the initial MALDI-ToF spectra (2 ± 2.35 SD). The decrease in peptide markers recognised in the MALDI-tims-Q-ToF was not expected but could be due to the freezer duration of approximately six months at -20°C between the mass spectrometry analyses as well as the already small amount of sampled material, among others. However, this is beyond anything that has been tested previously. A fair comparison between the two mass spectrometry instruments would require the original specimens to be resampled and analysed through the MALDI-tims-Q-ToF, instead of utilising the already gelatinised extracts, and at least involve similar storage durations between both types of mass spectrometry analysis.

The MALDI-ToF MS data have been uploaded to the repository Zenodo [77] (DOI: 10.5281/zenodo.10973909).

### Zooarchaeological analysis

Though the artefacts have lost most diagnostic features for morphological taxonomic identifications, it was in two cases possible, in combination with the ZooMS results, to further specify the species level. Sample CT_0009 identified as *Capra* sp. through ZooMS could be determined as *C*. *hircus* based on the size of the bone. Sample CT_0011 was identified as Cervidae through ZooMS, but could be specified further to *C*. *capreolus* through an additional assessment of the specimen.

Furthermore, all artefacts could be at least determined as the skeletal element of a long bone, including tibia, metapodial, metacarpus, and femur, except one whose element could not be identified (S2 Table).

## Discussion

In this study, it is evident that Cervidae and Caprinae long bones were primarily selected for the manufacturing of bone artefacts during the Early Neolithic at the site of Coro Trasito.

Taxonomic identifications could possibly have been required for some of the less burned specimens if sampled via bone chips [48] or utilising a second polishing film stick on the already sampled area [78] for ZooMS analysis increasing the taxonomic identification success rate. Concerning the burned artefacts, heat treatment of bone is known to break down collagen [79, 80], explaining the low taxonomic identification success rate and poor recovery of peptide markers of the heat-treated specimens. In fact, since burning or significant heat exposure is normally assumed to lead to complete proteomic degradation, the recovery of some peptide markers for such bone artefacts is noteworthy. Of the eight burned samples where at least 1 peptide marker was observed, the COL1α1 508–519 peptide was present, while the peptides COL1α2 978–990 and COL1α2 484–498 could be observed in respectively sample CT_0006 and CT_0012 (see S2 Table for more detail). Future research should therefore experimentally establish what the effect of heat treatment on bone proteome preservation is.

**Fig 8:**
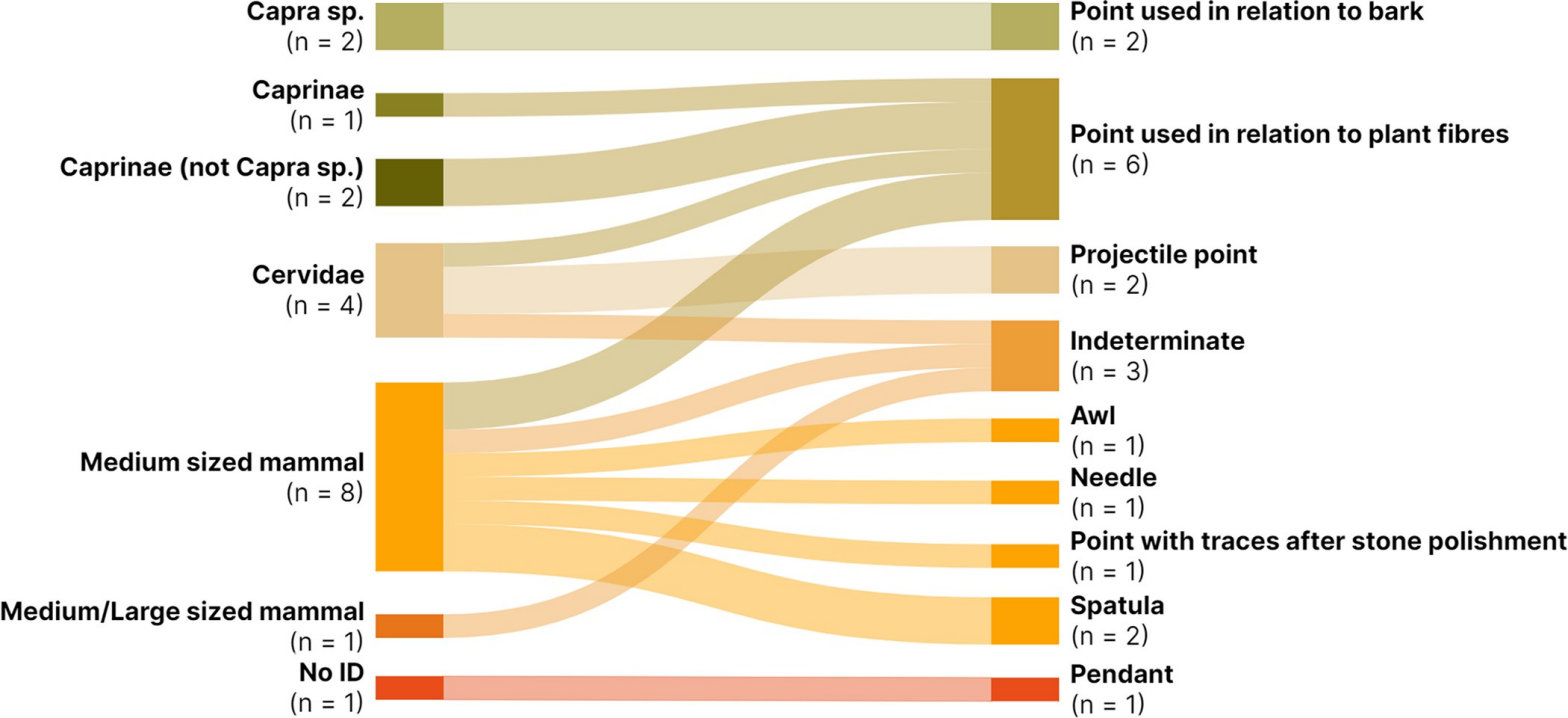
Alluvial plot illustrating the relationship between taxonomic identification and the artefact’s type/function. Each bone artefact is represented once on the left, indicating their taxonomic identity (ZooMS and bone morphology), and once on the right, indicating their object type (traceology). Three *C*. *elaphus* antler tools used in lithic production and one *S*. *scrofa* tusk used for the processing of plant fibres are not represented in the figure.

As for the taxonomically identified artefacts, both *Capra* sp. and Caprinae (not *Capra* sp.), most likely *C*. *hircus* and *O*. *aries* respectively, were selected respectively for the production of points used in relation to bark and possibly weaving plant fibres (e.g. cordage, textiles, and/or basketry) (Fig 8). Cervidae, including both *C*. *elaphus* and *C*. *capreolus*, were chosen for the production of projectile points, an indeterminate object, and a point used in relation to plant fibres. Interestingly, the latter was created from a *C*. *capreolus* metapodial resembling a more similar size group of Caprinae opposed to *C*. *elaphus*. On the taxonomic level of family, Cervidae seems to be related to a wider variety of artefact types, compared with that of Caprinae, involving weaponry and points used in handling plant fibres, but also objects associated with lithic production [43]. One *S*. *scrofa* tusk has also been identified as having been used in the processing of plant fibres [43]. However, the taxonomically identified portion of the specimen analysed counts for half of the object types present in the collection. The species selection for bone tool manufacture might therefore be more complex than seen here.

Nevertheless, our results stand in contrast to earlier studies, in which artefacts were morphologically taxonomically determined, where *O*. *aries* and *C*. *hircus* have the most prominent position within the production of osseous instruments, especially pointed tools. In the outer ranges of the pre-Pyrenees, at the Early Neolithic Cave of Chaves, located on the southern side of Sierra de Guara, approximately 100 km from Coro Trasito in bee-line [36], *O*. *aries* and *C*. *hircus* were predominately used in the production of bone objects, particularly awls [81]. This observation correlates with the surrounding non-artefact bone assemblage represented mostly by *O*. *aries* and *C*. *hircus* as well. Other parts of the Iberian Peninsula show similar species selection strategies for bone tool manufacture as at the Cave of Chaves based on morphological assessments. Neolithic sites in the Valencian Region revealed a predominance of small ruminant metapodials with over 90% of the identified specimens being associated with *O*. *aries* and *C*. *hircus*, and in some cases *C*. *capreolus*, based on the analysis of 411 awls. Metapodials were mainly selected from individuals over 3 years of age, ensuring the distal epiphysis was fused properly. Large ungulates constituted 6.2% of the assemblage, whereof 3.4% could be attributed to *C*. *elaphus* and 0.5% to *Equus* sp. [82]. However, in the Cave of l’Or, located close to Alicante, Valencia, *O*. *aries* and *C*. *hircus* still comprise the largest portion of the bone artefacts analysed, but a relatively high percentage of 23% of the bone objects could be identified as Cervidae, while no *S*. *domesticus* were utilised for the production of bone tools. Other species included in the production of bone artefacts included *Canis familiaris*, *V*. *vulpes*, largomorphs, Aves, and Piscis [83]. Further south, at the Neolithic site of Las Peñas de los Gitanos in Los Castillejos, Granada, the same distribution of *O*. *aries* and *C*. *hircus* is dominant, followed by, in descending order, *Sus* sp., *Bos* sp., Cervidae, and various carnivores, whereof domesticates presumably constituted the largest component [84].

A wide range of species selection strategies for bone tool manufacture thus seem to be present based on the various Iberian archaeological sites. Species selection strategies are likely influenced by fauna availability, properties of the skeletal elements, and the maturity of the animals for certain artefact types. In addition, hunting as a contributing economic factor and whichever social capital the activity might accommodate is a sincere notion. Hunting practices have been suggested to play an important role in early agricultural societies in the Iberian Peninsula [85, 86]. Moreover, graphic productions in the Levant region of Iberian Peninsula (e.g. Levantine and Schematic arts) suggest that hunting of big game played a significant role during the Neolithic, though there is no material evidence or dietary data that suggests a progressive dichotomy between livestock and wild game existed [87]. Pendants crafted from carnivore canine teeth also increase in numbers from the Early to the Late Neolithic throughout the Iberian Peninsula [82, 88]. The usage of Cervidae in the bone tool production, projectile points being exclusively made of this taxon at Coro Trasito, might therefore display an essential and standardised practice way of life, though maybe not habitual as the wild fauna bone portion constitutes around 10%, whereof around 5% can be attributed to Cervidae [34]. It is also worth speculating whether Cervidae and Cervinae, most likely *C*. *elaphus*, were used for the production of projectile points from long bones due to specific physical properties related to the species of *C*. *elaphus*, e.g. cortical thickness, and likewise for Caprinae and *C*. *Capreolus* in relation to points used in handling plant fibres. Micro-cracks in projectile bone points characteristic for stress related damage caused by bending forces can be detected, e.g. through tomograph imgaing [89, 90]. However, no study to our knowledge has assessed whether *C*. *elaphus* should be better suited for projectile points compared with the smaller *C*. *capreolus* or any Caprinae species.

With the exception of Cave of l’Or with a relative high amount of artefacts made from wild animals [83], the disproportionate ratio between Cervidae identifications attributed to artefacts and Cervidae identified as waste at Coro Trasito might not be a rare phenomenon. Bone artefacts from Dutch Neolithic sites have likewise shown the importance of incorporating *C*. *elaphus* in the bone tool manufacture despite *C*. *elaphus* generally decreasing in the subsistence economy [91]. Due to the difficulties in taxonomically identifying bone artefacts morphologically, data might be distorted and biased towards certain species [20, 92–94, among others]. The taxonomically unidentified bone artefact portion is typically not insignificant and this could be a potential risk in the assessment of the species composition of a bone artefact assemblage. Taxonomic identification methods like ZooMS thus have the significant capability to contribute to the study of bone artefacts.

Lastly, typological assessments of artefacts can be most helpful when exploring the relationship between the taxonomic origin and artefact type. Typologies can in many instances provide the function of the tool or object analysed, besides grouping the artefact’s cultural affiliation and temporal epoch. Moreover, traceological analyses can amplify the nuances related to our understanding of artefacts. Bone points might have similar shapes and sizes, but might have been used for entirely different purposes, while weaponry might have no traces of usage at all [95], perhaps indicating weapons being carried as a form of social capital or for ceremonial purposes [96, 97]. The intertwinement between technological, morphological, and proteomic analyses thus has the potential to expand our understanding of behavioural mechanisms related to bone tool manufacture, their usage, and their discard, both on a functional level as well as on an ontological level. Our case study at Coro Trasito stands as a testimony to the advantage of implementing traceological and ZooMS analyses alongside each other, as well as zooarchaeological assessments, on bone artefacts. Altogether, the approaches enable taxonomic identification of bone artefacts where diagnostic features are otherwise gone, identification of skeletal elements, taphonomics, and extensive knowledge on the usage of the bone artefacts, which combined allow for deeper insights into species selection strategies for the manufacture of bone objects and the tangible and intangible values of the animals used.

## Supporting information

S1 Table Data for building Fig 3. Faunal remains from Coro Trasito from layers referred to as NEO II, dating to approximately 4,900–4,700 cal BCE.
(PDF)

S2 Table Contextual, zooarchaeological, and proteomic data. Samples CT_0001-CT_0026 represent first MS analyses, while CT_0027-CT_0043 represents a selection for second MS analyses. See column RERUN_TWIN to relate first and second MS runs. Bone element abbreviations: FE = femur | LBD = long bone diaphysis | MTC = metacarpus | MTP = metapodial | ND = non-identified | T = tibia.
(PDF)
