# Supplementary material for: Combining traceological analysis and ZooMS on Early Neolithic bone artefacts from the cave of Coro Trasito, NE Iberian Peninsula: Cervidae used equally to Caprinae
Source: PLoS One. 2024 Jul 10;19(7):e0306448. doi: 10.1371/journal.pone.0306448 (PMC11236160; doi:10.1371/journal.pone.0306448)
Supplement: S1 Table — Faunal remains from Coro Trasito from layers referred to as NEO II, dating to approximately 4,900–4,700 cal BCE. (PDF) [file pone.0306448.s001.pdf]

Taxon	MNI	NISP
Bos taurus	3	8
Sus domesticus	6	26
Ovis aries	3	12
Capra hircus	2	2
O. aries/C. hircus	3	136
Cervus elaphus	1	10
Capreolus capre[illegible]	2	2
Capra pyrenaica	1	2
Sus scrofa	1	1
Oryctolagus cuni[illegible]	1	5
Vulpes vulpes	1	1
Ursus arctos	1	1

Species identified at Coro Trasito NEO II
