# Supplementary material for: Combining traceological analysis and ZooMS on Early Neolithic bone artefacts from the cave of Coro Trasito, NE Iberian Peninsula: Cervidae used equally to Caprinae
Source: PLoS One. 2024 Jul 10;19(7):e0306448. doi: 10.1371/journal.pone.0306448 (PMC11236160; doi:10.1371/journal.pone.0306448)
Supplement: S2 Table — Samples CT_0001-CT_0026 represent first MS analyses, while CT_0027-CT_0043 represents a selection for second MS analyses. See column RERUN_TWIN to relate first and second MS runs. Bone element abbreviations: FE = femur | LBD = long bone diaphysis | MTC = metacarpus | MTP = metapodial | ND = non-identified | T = tibia. (PDF) [file pone.0306448.s002.pdf]

ZooMS_ID	SITE	YEAR	OTHER	INVENTORY_No	RECORD_COORD_No	ARCHAEOL_LVL	CHRONOLOGY (Navarrete et al. 2023)	cal_BCE	COMMENTS	MATERIAL	TISSUE_TYPE	MORPH_TAXON_ID	BONE_ELEMENT
CT_0001	Coro Trasito	2019	19.41.A4A23.IO5	IO-05	19A-992	A-4A23				Bone	COR	M/L	T
CT_0002	Coro Trasito	2021	21.17.A4A37.IO1	IO-01	21A-779	A-4A37				Bone	COR	M	T
CT_0003	Coro Trasito	2019	19.41.A4A23.IO3	IO-03	19A-787	A-4A23				Bone	COR	M	MTC
CT_0004	Coro Trasito	2019	19.41.A4A23.IO4	IO-04	19A-833	A-4A23				Bone	COR	M	T
CT_0005	Coro Trasito	2021	21.17.A4A31.IO1	IO-01	21A-336	A-4A31 base				Bone	COR	M	MTP
CT_0006	Coro Trasito	2013	13.10.3006B.IO1	IO-01		3006B	NEO II/III	4999-4614	Carbon-14 date from ETH-88905	Bone	COR	M	LBD
CT_0007	Coro Trasito	2013	13.10.3005.IO4	IO-04		3005	NEO II/III			Bone	COR	M	LBD
CT_0008	Coro Trasito	2021	21.17.A4A31.IO2		21A-189	A-4A31 base				Bone	COR	M/L	FE
CT_0009	Coro Trasito	2022	22.43.A4B40b.IO1	IO-01	22A-559	A-4B40b				Bone	COR	Ovis/Capra	T
CT_0010	Coro Trasito	2021	21.17.A4A44.IO1	IO-01	21A-801	A-4A44				Bone	COR	M	LBD
CT_0011	Coro Trasito	2022	22.43.A4A54.IO1	IO-01	22A-658	A-4A54				Bone	COR	Capreolus capreolus	MTP
CT_0012	Coro Trasito	2013	13.10.3006.IO2	IO-02		3006	NEO II/III			Bone	COR	M/L	LBD
CT_0013	Coro Trasito	2013	13.10.3003.IO1	IO-01		3003	NEO II			Bone	COR	M	LBD
CT_0014	Coro Trasito	2021	21.17.A4A41.IO1	IO-01	FLO	A-4A41				Bone	COR	ND	ND
CT_0015	Coro Trasito	2022	22.43.A4A51.IO1	IO-01	22A-671	A-4A51				Bone	COR	M	LBD
CT_0016	Coro Trasito	2022	22.43.A4A46.IO1	IO-01	22A-036	A-4A46				Bone	COR	M	LBD
CT_0017	Coro Trasito	2013	13.10.3010.IO1	IO-01		3010	NEO II	4992-4786	Carbon-14 date from Beta-358571	Bone	COR	M	LBD
CT_0018	Coro Trasito	2013	13.10.3005.IO1	IO-01		3005	NEO II/III			Bone	COR	ND	ND
CT_0019	Coro Trasito	2019	19.41.A4A5.IO1	IO-01	19A-027	A-4A5				Bone	COR	M	LBD
CT_0020	Coro Trasito	2013	13.10.3006.IO1	IO-01		3006	NEO II/III			Bone	COR	M	FE
CT_0021	Coro Trasito	2013	13.10.3005.IO3	IO-03		3005	NEO II/III			Bone	COR	M	LBD
CT_0022	Coro Trasito	2013	13.10.3005.IO2	IO-02		3005	NEO II/III			Bone	COR	ND	ND
CT_0023	Coro Trasito	2013	13.10.3001.IO	IO		3001	Bronze Age			Bone	COR	ND	ND
CT_0024	Coro Trasito	2019	19.41.A3A47.IO1	IO-01		A-3A47	NEO II/III			Bone	COR	M	LBD
CT_0025	Coro Trasito	2013	13.10.3001.IO2			3001	Bronze Age			Bone	COR	ND	ND
CT_0026	Coro Trasito					Punzon Bronce 563	Bronze Age			Bone	COR	Canis	MTP
CT_0027	Coro Trasito												
CT_0028	Coro Trasito												
CT_0029	Coro Trasito												
CT_0030	Coro Trasito												
CT_0031	Coro Trasito												
CT_0032	Coro Trasito												
CT_0033	Coro Trasito												
CT_0034	Coro Trasito												
CT_0035	Coro Trasito												
CT_0036	Coro Trasito												
CT_0037	Coro Trasito												
CT_0038	Coro Trasito												
CT_0039	Coro Trasito												
CT_0040	Coro Trasito												
CT_0041	Coro Trasito												
CT_0042	Coro Trasito												
CT_0043	Coro Trasito												

ZooMS_ID	THERMAL_ALTERATION	THERMAL_STAGE (Stiner et al. 1995)	OTHER_OBSERVATIONS	ARTEFACT_TYPE	TRACE_FUNCTIONALITY	OTHER_TRACE_COMMENTS	SAMPLE_TISSUE_TYPE	RERUN_TWIN	RERUN_ORIGINAL
CT_0001	N	0		Projectile point	Weapon	Impact fracture?	COR		
CT_0002	N	0		Pointed tool	Used on bark		COR		
CT_0003	N	0		Bone point	Used on/in handling plants		COR		
CT_0004	N	0		Bone point	Used on/in handling plants		COR	CT_0027	O
CT_0005	N	0		Bone point	Used on/in handling plants	Possibly related to weaving plant fibers	COR		
CT_0006	Y	2		Needle	Used to perforate hides and/or skins		COR	CT_0028	O
CT_0007	Y	2		Awl	Used to perforate hides and/or skins	Fragmented	COR	CT_0029	O
CT_0008	Y	1		Not tool		Marrow extraction	COR		
CT_0009	N	0	Domesticate based on size	Bone point	Used on bark?		COR		
CT_0010	N	0		Projectile point	Weapon	Fragmented and polished	COR	CT_0030	O
CT_0011	N	0		Bone point	Used on/in handling plants	Related to weaving plant fibers	COR		
CT_0012	Y	5		Bone point?	Indetermiante	Impression fracture?	COR	CT_0031	O
CT_0013	N	0		Indeterminate		Biting marks and polishment similar to projectile points	COR		
CT_0014	?	0		Pendant	Decorative		COR	CT_0032	O
CT_0015	Y	3		Pointed tool	Used on/in handling vegetation	Related to weaving vegetation fibers	COR	CT_0033	O
CT_0016	Y	3		Pointed tool	Used on/in handling plants	Fractured during usage	COR	CT_0034	O
CT_0017	Y	2		Needle	Used to perforate hides and/or skins		COR	CT_0035	O
CT_0018	Y	3		Not tool			COR	CT_0036	O
CT_0019	Y	2		Indeterminate		Fragmented object with scrapings on both sides	COR	CT_0037	O
CT_0020	Y	2		Spatula?	Indeterminate		COR	CT_0038	O
CT_0021	Y	2		Spatula	Used in ceramic production		COR	CT_0039	O
CT_0022	Y	1		Not tool			COR		
CT_0023	N	0					COR	CT_0040	O
CT_0024	Y	2		Bone point		Traces after stone polishment	COR	CT_0041	O
CT_0025	Y	3					COR	CT_0042	O
CT_0026	N	0					COR	CT_0043	O
CT_0027								CT_0004	R
CT_0028								CT_0006	R
CT_0029								CT_0007	R
CT_0030								CT_0010	R
CT_0031								CT_0012	R
CT_0032								CT_0014	R
CT_0033								CT_0015	R
CT_0034								CT_0016	R
CT_0035								CT_0017	R
CT_0036								CT_0018	R
CT_0037								CT_0019	R
CT_0038								CT_0020	R
CT_0039								CT_0021	R
CT_0040								CT_0023	R
CT_0041								CT_0024	R
CT_0042								CT_0025	R
CT_0043								CT_0026	R

ZooMS_ID	SAMPLING_METHOD	EXTRACTION_PROTOCOL	PLATE_TUBE	WELL_PLATE_No	WELL_PLATE_LOCATION	RUN	SPOT_1	SPOT_2	SPOT_3	MS_INSTRUMENT	BARCODE_MOPRH_ZooMS_COMBO
CT_0001	PF	AmBic	Tube			20230920_MMA_Jakob_20230907_HN02_1005467	F8	F11	F14	MALDI-ToF	Cervinae
CT_0002	PF	AmBic	Tube			20230920_MMA_Jakob_20230907_HN02_1005467	F9	F12	F15	MALDI-ToF	Capra sp.
CT_0003	PF	AmBic	Tube			20230920_MMA_Jakob_20230907_HN02_1005467	F10	F13	F16	MALDI-ToF	Caprinae (not Capra sp.)
CT_0004	PF	AmBic	Tube			20230920_MMA_Jakob_20230907_HN02_1005467	F17	F20	F23	MALDI-ToF	Caprinae
CT_0005	PF	AmBic	Tube			20230920_MMA_Jakob_20230907_HN02_1005467	F18	F21	F24	MALDI-ToF	Caprinae (not Capra sp.)
CT_0006	PF	AmBic	Tube			20230920_MMA_Jakob_20230907_HN02_1005467	F19	F22	G1	MALDI-ToF	No ID
CT_0007	PF	AmBic	Tube			20230920_MMA_Jakob_20230907_HN02_1005467	G2	G5	G8	MALDI-ToF	No ID
CT_0008	PF	AmBic	Tube			20230920_MMA_Jakob_20230907_HN02_1005467	G3	G6	G9	MALDI-ToF	Cervinae
CT_0009	PF	AmBic	Tube			20230920_MMA_Jakob_20230907_HN02_1005467	G4	G7	G10	MALDI-ToF	Capra hircus
CT_0010	PF	AmBic	Tube			20230920_MMA_Jakob_20230907_HN02_1005467	G11	G14	G17	MALDI-ToF	Cervidae
CT_0011	PF	AmBic	Tube			20230920_MMA_Jakob_20230907_HN02_1005467	G12	G15	G18	MALDI-ToF	Capreolus capreolus
CT_0012	PF	AmBic	Tube			20230920_MMA_Jakob_20230907_HN02_1005467	G13	G16	G19	MALDI-ToF	No ID
CT_0013	PF	AmBic	Tube			20230920_MMA_Jakob_20230907_HN02_1005467	G20	G23	H3	MALDI-ToF	Cervidae
CT_0014	PF	AmBic	Tube			20230920_MMA_Jakob_20230907_HN02_1005467	G21	G24	H4	MALDI-ToF	No ID
CT_0015	PF	AmBic	Tube			20230920_MMA_Jakob_20230907_HN02_1005467	G22	H1	H6	MALDI-ToF	No ID
CT_0016	PF	AmBic	Tube			20230920_MMA_Jakob_20230907_HN02_1005467	H7	H12	H16	MALDI-ToF	No ID
CT_0017	PF	AmBic	Tube			20230920_MMA_Jakob_20230907_HN02_1005467	H9	H13	H18	MALDI-ToF	No ID
CT_0018	PF	AmBic	Tube			20230920_MMA_Jakob_20230907_HN02_1005467	H10	H15	H19	MALDI-ToF	No ID
CT_0019	PF	AmBic	Tube			20230920_MMA_Jakob_20230907_HN02_1005467	H21	I1	I4	MALDI-ToF	No ID
CT_0020	PF	AmBic	Tube			20230920_MMA_Jakob_20230907_HN02_1005467	H22	I2	I5	MALDI-ToF	No ID
CT_0021	PF	AmBic	Tube			20230920_MMA_Jakob_20230907_HN02_1005467	H24	I3	I6	MALDI-ToF	No ID
CT_0022	PF	AmBic	Tube			20230920_MMA_Jakob_20230907_HN02_1005467	I7	I10	I13	MALDI-ToF	Cervidae
CT_0023	PF	AmBic	Tube			20230920_MMA_Jakob_20230907_HN02_1005467	I8	I11	I14	MALDI-ToF	No ID
CT_0024	PF	AmBic	Tube			20230920_MMA_Jakob_20230907_HN02_1005467	I9	I12	I15	MALDI-ToF	No ID
CT_0025	PF	AmBic	Tube			20230920_MMA_Jakob_20230907_HN02_1005467	I16	I19	I22	MALDI-ToF	No ID
CT_0026	PF	AmBic	Tube			20230920_MMA_Jakob_20230907_HN02_1005467	I17	I20	I23	MALDI-ToF	Canis
CT_0027	PF	AmBic	Tube+Plate	13	A1	JH12_1007106_Files	0	1	2	MALDI tims-Q-ToF	
CT_0028	PF	AmBic	Tube+Plate	13	A2	JH12_1007106_Files	3	4	5	MALDI tims-Q-ToF	
CT_0029	PF	AmBic	Tube+Plate	13	A3	JH12_1007106_Files	6	7	8	MALDI tims-Q-ToF	
CT_0030	PF	AmBic	Tube+Plate	13	A4	JH12_1007106_Files	9	10	11	MALDI tims-Q-ToF	
CT_0031	PF	AmBic	Tube+Plate	13	A5	JH12_1007106_Files	12	13	14	MALDI tims-Q-ToF	
CT_0032	PF	AmBic	Tube+Plate	13	A6	JH12_1007106_Files	15	16	17	MALDI tims-Q-ToF	
CT_0033	PF	AmBic	Tube+Plate	13	A7	JH12_1007106_Files	18	19	20	MALDI tims-Q-ToF	
CT_0034	PF	AmBic	Tube+Plate	13	A8	JH12_1007106_Files	21	22	23	MALDI tims-Q-ToF	
CT_0035	PF	AmBic	Tube+Plate	13	A9	JH12_1007106_Files	24	26	27	MALDI tims-Q-ToF	
CT_0036	PF	AmBic	Tube+Plate	13	A10	JH12_1007106_Files	28	29	30	MALDI tims-Q-ToF	
CT_0037	PF	AmBic	Tube+Plate	13	A11	JH12_1007106_Files	31	32	33	MALDI tims-Q-ToF	
CT_0038	PF	AmBic	Tube+Plate	13	A12	JH12_1007106_Files	34	35	36	MALDI tims-Q-ToF	
CT_0039	PF	AmBic	Tube+Plate	13	B1	JH12_1007106_Files	37	38	39	MALDI tims-Q-ToF	
CT_0040	PF	AmBic	Tube+Plate	13	B2	JH12_1007106_Files	40	41	42	MALDI tims-Q-ToF	
CT_0041	PF	AmBic	Tube+Plate	13	B3	JH12_1007106_Files	43	44	45	MALDI tims-Q-ToF	
CT_0042	PF	AmBic	Tube+Plate	13	B4	JH12_1007106_Files	46	47	48	MALDI tims-Q-ToF	
CT_0043	PF	AmBic	Tube+Plate	13	B5	JH12_1007106_Files	49	50	51	MALDI tims-Q-ToF	

ZooMS_ID	BARCODE_ID	BARCODE_ID_BROAD	COL1α1 508-519	COL1α2 978-990	COL1α2 484-498	COL1α2 502-519	COL1α2 292-309	COL1α2 793-816	COL1α2 454-483	COL1α1 586-618	COL1α2 757-789	PEPTIDE_MARKER_No	POST_MS_NOTES
CT_0001	Cervinae	Cervidae	1105.6	1180.6+1196.6	1427.7	1550.8	1648.8	2131.1	2792.4	2883.4	3017.4+3033.4	9	
CT_0002	Capra sp.	Caprinae	1105.6	1180.6+1196.6	1427.7	1580.8	1648.8	2131.1	2792.4	2883.4+2899.4	3093.4	9	
CT_0003	Caprinae (not Capra sp.)	Caprinae	1105.6	1180.6+1196.6	1427.7	1580.8	1648.8	2131.1	2792.4	2883.4	3017.4+3033.4	9	
CT_0004	Caprinae	Caprinae	1105.6	1196.6	1427.7	1580.8	1648.8	2131.1	x	x	x	6	
CT_0005	Caprinae (not Capra sp.)	Caprinae	1105.6	1180.6+1196.6	1427.7	1550.8	1648.8	2131.1	2792.4	2883.4+2899.4	3017.4+3033.4	9	
CT_0006	No ID	No ID	1105.6	x	1427.7	x	x	x	x	x	x	2	
CT_0007	No ID	No ID	1105.6	x	x	x	x	x	x	x	x	1	
CT_0008	Cervinae	Cervidae	1105.6	1196.6	1427.7	1550.8	1648.8	2131.1	2792.4	2883.4+2899.4	3017.4+3033.4	9	
CT_0009	Capra sp.	Caprinae	1105.6	1180.6+1196.6	1427.7	1580.8	1648.8	2131.1	x	2883.4	3093.4	9	
CT_0010	Cervidae	Cervidae	1105.6	1180.6+1196.6	1427.7	1550.8	1648.8	2131.1	2792.4	2883.4+2899.4	x	8	
CT_0011	Cervidae	Cervidae	1105.6	1180.6+1196.6	1427.7	1550.8	1648.8	2131.1	x	2883.4+2899.4	x	7	
CT_0012	No ID	No ID	1105.6	1180.6	x	x	x	x	x	x	x	2	1.723783215
CT_0013	Cervidae	Cervidae	1105.6	1196.6	1427.7	1550.8	1648.8	2131.1	x	x	x	6	
CT_0014	No ID	No ID	1105.6	1180.6+1196.6	x	x	x	x	x	x	x	2	1.414213562
CT_0015	No ID	No ID	1105.6	x	x	x	x	x	x	x	x	1	
CT_0016	No ID	No ID	1105.6	x	x	x	x	x	x	x	x	1	
CT_0017	No ID	No ID	1105.6	x	x	x	x	x	x	x	x	1	
CT_0018	No ID	No ID	1105.6	x	x	x	x	x	x	x	x	1	
CT_0019	No ID	No ID	1105.6	x	x	x	x	x	x	x	x	1	
CT_0020	No ID	No ID	1105.6	x	x	x	x	x	x	x	x	1	
CT_0021	No ID	No ID	x	x	x	x	x	x	x	x	x	0	
CT_0022	Cervidae	Cervidae	x	x	1427.7	1550.8	1648.8	2131.1	x	2883.4+2899.4	x	5	
CT_0023	No ID	No ID	x	x	x	x	x	x	x	x	x	0	
CT_0024	No ID	No ID	x	x	x	x	x	x	x	x	x	0	
CT_0025	No ID	No ID	x	x	x	x	x	x	x	x	x	0	
CT_0026	Canis	Carnivora	x	x	1453.7	x	x	2131.1	x	2853.4	x	3	
CT_0027	Caprinae	Caprinae	1105.6	x	1427.7	1580.8	1648.8	2131.1	x	2883.4	x	6	
CT_0028	No ID	No ID	1105.6	x	x	x	x	x	x	x	x	1	
CT_0029	No ID	No ID	x	x	x	x	x	x	x	x	x	0	
CT_0030	Cervidae	Cervidae	1105.6	x	1427.7	1550.8	1648.8	2131.1	x	2883.4+2899.4	x	6	
CT_0031	No ID	No ID	x	x	x	x	x	x	x	x	x	0	
CT_0032	No ID	No ID	x	x	x	x	x	x	x	x	x	0	
CT_0033	No ID	No ID	1105.6	x	x	x	x	x	x	x	x	1	
CT_0034	No ID	No ID	x	x	x	x	x	x	x	x	x	0	
CT_0035	No ID	No ID	x	x	x	x	x	x	x	x	x	0	
CT_0036	No ID	No ID	x	x	x	x	x	x	x	x	x	0	
CT_0037	No ID	No ID	x	x	x	x	x	x	x	x	x	0	
CT_0038	No ID	No ID	x	x	x	x	x	x	x	x	x	0	
CT_0039	No ID	No ID	x	x	x	x	x	x	x	x	x	0	
CT_0040	No ID	No ID	x	x	x	x	x	x	x	x	x	0	
CT_0041	No ID	No ID	x	x	x	x	x	x	x	x	x	0	
CT_0042	No ID	No ID	x	x	x	x	x	2131.1	x	x	x	0	
CT_0043	Canis	Carnivora	1105.6	x	1453.7	x	x	2131.1	x	2853.4+2869.4	x	4	

ZooMS_ID	P836	P1105	P1690	P1706	P1836	P2056	P2072	P2088	P2104	P2689	P2705
CT_0001		0.78									
CT_0002		0.83									0.88
CT_0003		0.77									
CT_0004											
CT_0005		0.82									
CT_0006											
CT_0007											
CT_0008											0.77
CT_0009		0.82									
CT_0010		0.81									
CT_0011		0.74									
CT_0012											
CT_0013		0.6									
CT_0014											
CT_0015											
CT_0016											
CT_0017											
CT_0018											
CT_0019											
CT_0020											
CT_0021											
CT_0022											
CT_0023											
CT_0024											
CT_0025											
CT_0026											
CT_0027											
CT_0028											
CT_0029											
CT_0030											
CT_0031											
CT_0032											
CT_0033											
CT_0034											
CT_0035											
CT_0036											
CT_0037											
CT_0038											
CT_0039											
CT_0040											
CT_0041											
CT_0042											
CT_0043											
